# First report of full colon transplantation as part of a visceral allograft

**DOI:** 10.1007/s10151-025-03115-5

**Published:** 2025-03-07

**Authors:** R. J. Cruz, C. Powers, V. Gunabushanam, A. Khanna

**Affiliations:** 1 Gastrointestinal Rehabilitation and Transplant Center - GIRTC, Starzl Transplantation Institute, Pittsburgh, PA USA; 2 Department of Surgery, University of Pittsburgh School of Medicine, Pittsburgh, PA USA

**Keywords:** Colon transplant, Intestinal transplant, Multivisceral transplantation, Intestinal ischemia, Short gut syndrome, Parenteral nutrition, TPN

## Abstract

**Background:**

The inclusion of the colon as part of intestinal and multivisceral allografts has increased in the last decade.

**Methods:**

We describe for the first time in the literature a full colon transplantation as a part of a visceral allograft. The new approach involves modifications of the procurement technique with preservation of all three visceral aortic branches and incorporation of the descending and sigmoid colon as a part of the allograft.

**Result:**

Seventeen months after transplantation, the patient is off any parenteral nutritional support, on full oral nutrition without the need for a single antidiarrheal agent.

**Conclusion:**

The introduction of this novel technique could open new opportunities for hindgut reconstruction for patients requiring visceral transplantation, with potential increase in allograft absorptive capacity and improvement in quality of life.

## Introduction

The inclusion of the colon as part of intestinal and multivisceral allografts was once associated with increased rejection rates, bacterial translocation, and graft loss [1, 2]. However, recent studies have shown that including the colon as part of the intestinal allograft improves/confers renal protection and increases ileostomy reversal rates, with no changes in acute cellular rejection and/or mortality rates [3–5]. In a recent retrospective analysis of data registered in the United Network for Organ Sharing (UNOS)/Organ Procurement and Transplantation Network (OPTN) database, Matsushima et al. reported a steady increase in the inclusion of the colon as part of the intestinal allograft in the last decade [3].

The ascending colon with or without the transverse colon is most commonly used as part of an allograft. We herein describe a new technique for full colon transplantation during multivisceral transplantation. The new approach involves few modifications of the procurement technique, with preservation of all three visceral aortic branches and the inclusion of the descending and sigmoid colon as a part of the allograft. The inclusion of the full colon as part of a multivisceral allograft has the potential to improve absorptive functions and quality of life, particularly in patients requiring visceral transplantation who have previously undergone total or near total colectomy or who present significant motility disorders of the colon.

## Case report

### Case material

On April 8, 2023, a 64-year-old woman with ultrashort bowel syndrome underwent multivisceral transplantation for irreversible intestinal failure. In October 2021, she was transferred to our institution from an out-of-state facility for extensive mesenteric ischemia. The patient has a history of hyperlipidemia, diabetes, and hypertension, with significant atherosclerosis involving the aorta and its visceral branches. The patient underwent total enterectomy, extended right colectomy, primary closure of the duodenal stump and gastrocolic anastomosis, and required long-term parenteral nutrition support at home thereafter. During the pre-transplantation evaluation, the left colon was found to be ischemic with a significant arteriosclerotic burden on the inferior mesenteric artery (IMA), which was the only vessel supplying the remaining colon. Liver biopsy revealed chronic steatohepatitis with mild periportal fibrosis (SAF score S3A3F2). Notably, the patient underwent extended left hepatectomy in 2015 for cholangiocarcinoma. After 340 days on the list, appropriately sized organs from a 27-year-old deceased donor who weighed 50 kg and had the same blood type became available. The human leukocyte antigen (HLA) match was random, with a negative lymphocytotoxic crossmatch. University of Wisconsin solution was used for preservation. Immunosuppression was tacrolimus-based, and the recipient received a total of 7.5 mg/kg of Thymoglobulin (Genzyme, Cambridge, MA). Maintenance steroids were added to the tacrolimus monotherapy. Postoperative management, including antimicrobial prophylaxis, nutritional care, and endoscopic surveillance with random mucosal biopsies of both the colon and small bowel, has been described elsewhere [6].

### Donor surgery

**Fig. 1:**
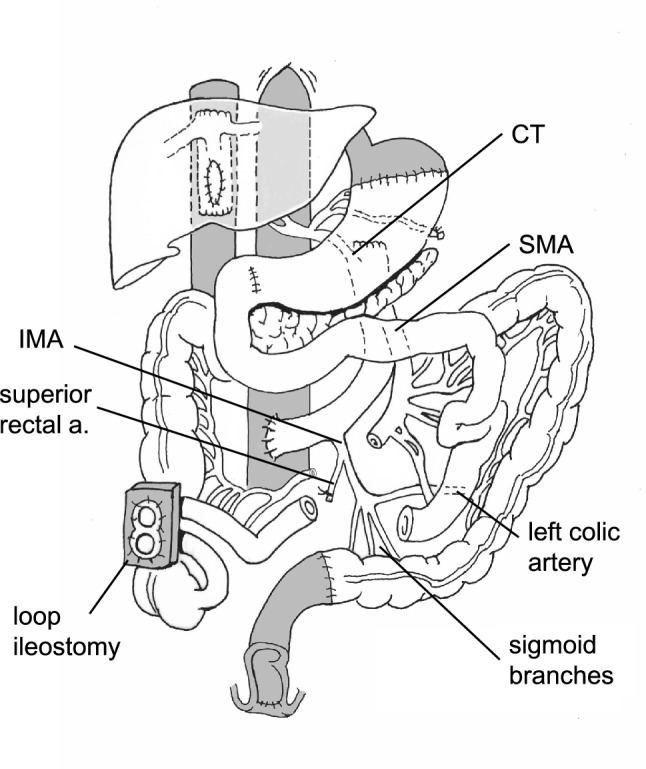
Illustration of vascular and gastrointestinal reconstruction after full multivisceral transplantation. Note the aortic conduit with preservation of all three visceral branches. All the inferior mesenteric artery branches are preserved except for the superior rectal artery

**Fig. 2:**
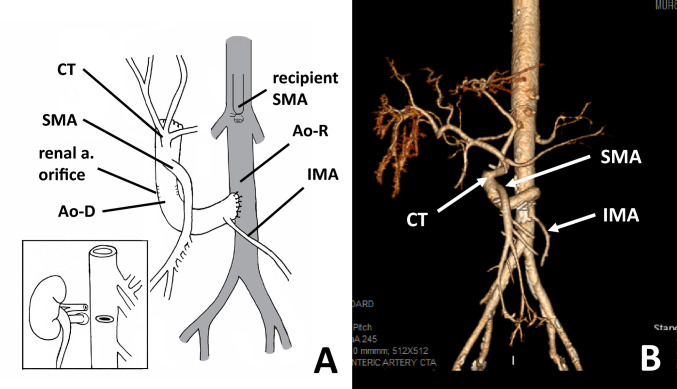
**a** Vascular reconstruction with preservation of all three main visceral branches, including the celiac trunk (CT), superior mesenteric artery (SMA), and inferior mesenteric artery (IMA). The renal artery orifices and the proximal aortic stump were oversewn. Vascular inflow was complete with an end-to-side anastomosis between the donor and recipient aortae (Ao-D and Ao-R, respectively). **b** Three-dimensional reconstruction from the CT angiogram showing preservation of all three aortic visceral branches

The organs were harvested by our team by using a previously described technique. All the blood supply to the colon, including the ileocolic, right, middle, and left colic arteries as well as the inferior mesenteric artery, were preserved (Fig. 1). During procurement, the mesocolon and the colonic arterial arcades were meticulously dissected and preserved. All the IMA branches were preserved except for the superior rectal artery, which was ligated (Fig. 1). The gastroesophageal junction and the colon at the level of the distal sigmoid were transected with a gastrointestinal stapler. The abdominal aorta of the donor was isolated from the hiatus all the way down to its bifurcation, and all three main visceral branches, including the celiac trunk, superior mesenteric artery, and inferior mesenteric artery, were preserved. During kidney procurement, the continuity of the aorta was preserved, and small patches from the aorta were resected and retained as a part of the renal artery allografts (Fig. 2a, insert). The multivisceral allograft, including the stomach, duodenum, pancreas, small bowel, and entire colon, was retrieved en bloc.

### Recipient operation and back table preparation

Through an extended midline incision, the abdominal cavity was entered, and the cavity was fully explored. As expected, the left colon appeared to be ischemic. We then proceeded with abdominal evisceration as previously described by our group. During evisceration, we completed the colectomy and transected the native colon at the level of the distal sigmoid. The infrarenal aorta was then isolated, and the right hepatic vein was transected with an endovascular stapler. The back table was completed with oversewing the proximal supraceliac aortic stump, as well as the previous renal artery orifices on the donor aorta (Fig. 2a, b). The multivisceral allograft was then brought to the field, and reconstitution of the vascular blood supply to the visceral allograft was established by an end-to-side anastomosis, as shown in Fig. 2. There was a mild ischemia–reperfusion injury with minimal use of inotropic support in the early stage after reperfusion. The cold ischemia time was 4 h 32 min, and the warm ischemia time was 33 min. After allograft revascularization, a gastrogastrostomy in two layers was completed, followed by pyloroplasty. We then turned our attention to the hindgut anastomosis. A hand-sewn two-layer anastomosis between the sigmoid allograft and the recipient’s distal sigmoid was then created. After ascertaining restoration of good blood flow throughout the colon, a diverting loop ileostomy was created 25 cm proximal to the ileocecal valve. The appendix was removed, and the abdomen was closed.

### Clinical course

There were no obvious technical post-transplant complications. However, her postoperative course was complicated by end-stage renal disease (ESRD) requiring renal replacement therapy. The cause of ESRD was multifactorial, including sepsis, drug-related nephrotoxicity, and pre-transplant borderline renal function due to diabetes and hypertension. Three months post-transplant the patient developed lung aspergillosis, requiring full treatment with voriconazole for 24 weeks, delaying the already planned ostomy closure. After resolution of her medical problems, the patient was discharged to inpatient rehabilitation with no further medical or immunologic complications. Seventeen months after transplantation, the patient is off any parenteral nutritional support, on full oral nutrition without the need for a single antidiarrheal agent. The patient is currently on tacrolimus monotherapy with a 12-h target trough level of 3–5 ng/dL. Recent surveillance biopsies of both the small bowel and colon continued to show intact architecture with no evidence of rejection. Unfortunately, the patient is still on renal replacement therapy and has been worked-up for living donor kidney transplantation.

## Discussion

To our knowledge, this is the first report of full colon transplant as part of a visceral allograft. This report demonstrated the adequacy of blood supply to the distal colon, including the distal sigmoid, via the donor inferior mesenteric artery. Technical innovations have been introduced over the last two decades to the originally described multivisceral transplant, including the inclusion of the kidneys, esophagus, and bladder segment [7–10]. However, no modifications have been made regarding the colonic allograft. Most colon-containing allografts include the right and transverse colon with a blood supply from the ileocolic, right and sometimes middle colic arteries. Even when lower anastomosis to the rectum are required, the right or transverse colon allografts are used for anastomosis [4, 5]. In 2010, we introduced an innovative procedure for hindgut reconstruction during a small bowel transplant. The technique involves a sphincter-preserving pull-through anastomosis of the colon to the anal verge [11]. Even in this case, the proximal descending colon was used for the colo-anal anastomosis.

Preoperative imaging studies and colonoscopy suggested some degree of colonic ischemia; therefore, resection of the descending native colon was anticipated. Two options for preserving the IMA as a part of the allograft were discussed before procurement. First, both kidneys were procured via resection of small aortic patches and closure of the aortic renal orifices at the back table (insert Fig. 2a). The second option was resection of an aortic ring containing both renal arteries (giving the renal transplant teams a larger patch) and anastomosis of the aortic conduit during benching. Fortunately, we were able to use the first technique avoiding additional anastomosis during the back table. Direct anastomosis of the distal donor abdominal aorta to the recipient infrarenal aorta, without the creation of Carrel’s patch, was previously described by several groups [12]. This approach facilitates allograft implantation while reducing the surgical time and the complexity of vascular reconstruction during back table.

The incorporation of entire colon as part of the allograft can be utilized in both full and modified multivisceral transplantation. We believe that the inclusion of the entire colon during transplantation can be a valuable option in two specific situations. First, patients with familial adenomatous polyposis with limited disease in the rectum and mutations proximal to the 1250 codon who underwent total colectomy with ileorectal anastomosis in the early stages of life. Second, patients (usually female) who underwent total colectomy and are at high risk for fecal incontinence with a transverse colon-to-rectal anastomosis due to loose stools.

In summary, the novel technique described herein could open new opportunities for hindgut reconstruction for patients requiring visceral transplantation. Ensuring a robust blood supply to the donor descending and sigmoid colon via an uninterrupted IMA allows a well-vascularized distal colon allograft. Additional studies with a larger sample of patients are necessary to evaluate the potential increase in allograft absorptive capacity, the extent of improvement in quality of life, and the likelihood of discontinuation of antidiarrheal medications in patients who have undergone transplantation of the entire colon.

## Data Availability

No datasets were generated or analysed during the current study.

## Abbreviations

ESRD: End-stage renal disease
GE: Gastroesophageal
HLA: Human leukocyte antigen
IMA: Inferior mesenteric artery
OPTN: Organ Procurement and Transplantation Network
UNOS: United Network for Organ Sharing

## References

[1] Todo S, Reyes J, Furukawa H et al (1995) Outcome analysis of 71 clinical intestinal transplantations. Ann Surg 222(3):270–80 (discussion 80-2) 7677458 10.1097/00000658-199509000-00006 PMC1234805

[2] Raofi V, Holman DM, Dunn TB et al (1999) Comparison of rejection rate and functional outcome of small bowel transplantation alone or in conjunction with the ileocecal valve versus combined small and large bowel transplantation. Clin Transplant 5:389–94 10.1034/j.1399-0012.1999.130504.x 10515219

[3] Matsushima H, Sasaki K, Nair A et al (2024) The impact of colonic allograft inclusion on intestinal transplantation outcomes: results from UNOS/OPTN database analysis. Clin Transplant 38(1):e15213 38064299 10.1111/ctr.15213

[4] Ewald C, Swanson BJ, Vargas L et al (2020) Including colon in intestinal transplantation: a focus on post-transplant renal function - a retrospective study. Transpl Int 33(2):142–8 31523865 10.1111/tri.13523

[5] Kato T, Selvaggi G, Gaynor JJ et al (2008) Inclusion of donor colon and ileocecal valve in intestinal transplantation. Transplantation 86(2):293–297 18645493 10.1097/TP.0b013e31817ef01c

[6] Abu-Elmagd KM, Costa G, Bond GJ et al (2009) Five hundred intestinal and multivisceral transplantations at a single center: major advances with new challenges. Ann Surg 250(4):567–581 19730240 10.1097/SLA.0b013e3181b67725

[7] Vakili K, Kim HB (2014) Partial esophageal transplantation is possible as part of a multivisceral graft. Am J Transpl 14(3):720–3 10.1111/ajt.12623 24447794

[8] Cruz RJ Jr, Costa G, Bond GJ et al (2011) Modified multivisceral transplantation with spleen-preserving pancreaticoduodenectomy for patients with familial adenomatous polyposis “Gardner’s syndrome.” Transplantation 91(12):1417–1423 21512435 10.1097/TP.0b013e31821ab93b

[9] Cruz RJ Jr, Costa G, Bond G et al (2010) Modified “liver-sparing” multivisceral transplant with preserved native spleen, pancreas, and duodenum: technique and long-term outcome. J Gastroint Surg Tract 14(11):1709–1721 10.1007/s11605-010-1317-5 20844978

[10] de Oliveira Kunzler, Maia F, Tekin A et al (2020) Use of pediatric donor en bloc kidneys along with bladder segment in pediatric liver-kidney and multivisceral-kidney transplantation. Pediatr Transplant 24(1):e13596 31605438 10.1111/petr.13596

[11] Eid KR, Costa G, Bond GJ et al (2010) An innovative sphincter preserving pull-through technique with en bloc colon and small bowel transplantation. Am J Transplant 10(8):1940–6 20636461 10.1111/j.1600-6143.2010.03167.x

[12] Nishida S, Vaidya A, Kato T, Nakamura N, Madariaga J, Tzakis A (2004) Use of donor aorta for arterial reconstruction in paediatric liver and multivisceral transplantation. Br J Surg 91(6):705–708 15164438 10.1002/bjs.4550

